# Robust behavioural effects in response to acute, but not repeated, terpene administration in Zebrafish (*Danio rerio*)

**DOI:** 10.1038/s41598-021-98768-1

**Published:** 2021-09-28

**Authors:** Joshua Szaszkiewicz, Shannon Leigh, Trevor J. Hamilton

**Affiliations:** 1 Department of Psychology, MacEwan University, 6-366, 10700 – 104 Ave. NW, Edmonton, AB T5J 4S2 Canada; 2 Neuroscience and Mental Health Institute, University of Alberta, Edmonton, AB T6G 2H7 Canada

**Keywords:** Animal behaviour, Neuroscience

## Abstract

Terpenes are fragrant aromatic compounds produced by a variety of plants, most notably cannabis and hops. With increasing legalization of cannabis there is a need to better understand the behavioural effects of terpenes and ultimately their therapeutic value. Our study investigated the dose-dependent impact of three terpenes (limonene 0.25, 0.5, 0.75%; β-myrcene 0.001, 0.01, 0.1%; and 0.0001, 0.001, 0.00125% linalool) on zebrafish (*Danio rerio*) behaviour when exposed both acutely and repeatedly over a 7-day period. Anxiety-like behaviour, boldness, and locomotion were assessed using the open field test and the novel object approach test. In the acute dosing experiment, limonene and β-myrcene exposed groups demonstrated a significant decrease in locomotion, a decrease in anxiety-like behaviour, and an increase in boldness, while linalool treatment groups demonstrated only minor alterations in locomotion. Moreover, repeated exposure to limonene (0.39%) or β-myrcene (0.0083%) for a seven day period did not result in any significant behavioural effects. In conclusion, our study provides support for an anxiolytic and sedative effect in zebrafish in response to acute limonene and β-myrcene exposure that is no longer present after one week of repeated exposure.

## Introduction

For centuries, cannabis has been used medicinally for its analgesic, sedative, and anticonvulsive effects^1,2^. Cannabis is now legal in some countries like Canada, and it is necessary to determine the actions of its phytochemical components, as well as their potential for therapeutic treatment. While a wealth of studies have demonstrated the potential health benefits of cannabinoid products like THC and CBD^3–7^, the therapeutic value of terpenes, which are also present in cannabis, have received significantly less attention^8^. Terpenes are a diverse group of phytochemicals that give plants their aroma and are a significant component in plant resin and essential oils. Over 200 terpenes have been identified in the cannabis plant which primarily consist of monoterpenoids, containing a 10-carbon backbone, and sesquiterpenoids which have a 15-carbon backbone^9,10^.

Terpenes have been shown to possess a wide range of medicinal properties including anti-inflammatory, anxiolytic, antiviral, antifungal, antibacterial, and anti-cancer effects in human and murine models^2,10–13^ and therefore may play an integral role in producing therapeutic effects observed in medicinal cannabis. Scientific processes are used develop standardized ‘strains’ of cannabis that contain specific proportions of cannabinoids and terpenes and accurately test for their levels^12^. Previous research has found numerous pharmacological effects in a variety of terpenes^2,10–14^, however, the study of behavioural effects of terpenes is only in its infancy. The monoterpenoids β-myrcene, limonene, and linalool are among the most common terpenes found in cannabis^15^, and were the focus of the current study.

β-Myrcene is an effective analgesic in murine studies. Lorenzetti et al. (1991) induced hyperalgesia in mice via injections of prostaglandin E2 and found that β-myrcene significantly reduced the intensity of the hyperalgesia, suggesting a strong analgesic effect^16^. Furthermore, in contrast to morphine, mice that were repeatedly dosed with β-myrcene over a period of five days did not develop any tolerance^16^. Similarly, Rao et al. (1990) induced hyperalgesia in mice using the hot plate method and found that preparations of β-myrcene significantly reduced nociception^17^. Treatment with naloxone prior to the hot plate test reversed the anti-nociceptive effect of β-myrcene, suggesting a potential opioid-related mechanism of action. Studies have also provided support for a sedative and anesthetic effect of β-myrcene^18–20^. Mice dosed with β-myrcene demonstrated significantly increased muscle relaxation and lengthened barbiturate-induced sleeping time compared to controls^18^. Furthermore, β-myrcene was determined to be an effective anaesthetic agent in rainbow trout (*Oncorhynchus mykiss*)^20^ and in common carp (*Cyprinus carpio*)^19^. β-Myrcene in cannabis may contribute to the heavy physical sedation felt by cannabis consumers which is referred to as “couch-lock”^13^.

Linalool is a monoterpenoid alcohol that is predominantly found in essential oil extracts of lavender, hops, and various cannabis strains. Linalool has demonstrated sedative, anxiolytic, and anti-inflammatory properties^13^, and therefore may possess therapeutic efficacy. Buchbauer et al. (1993) tested fragrant compounds and their essential oils on mice locomotion and found that inhalation of linalool decreased motility, suggesting a strong sedative effect^21^. Moreover, Souto-Maior et al. (2011) found that inhalation of linalool oxide at a variety of concentrations significantly decreased anxiety-like behaviour assessed in the elevated-plus maze and light/dark test^22^. The anxiolytic effect of linalool in mice was further supported by Linck et al. (2010) who observed decreased anxiety-like behaviour in the light/dark test in addition to decreased aggressive behaviour^23^. Linalool has also demonstrated an anti-inflammatory effect, as mice given linalool prior to cigarette smoke exposure were protected against lung inflammation compared to mice who were exposed solely to cigarette smoke^24^.

Limonene is the predominate terpene in citrus essential oil extracts and various cannabis strains such as “Girl Scout Cookie, Purple Kush, and Chocolope”^2,25^. In murine studies, limonene has demonstrated consistent anxiolytic effects in multiple behavioural tests of anxiety-like behaviour such as the elevated plus maze, open field, and light/dark test^26–28^. Moreover, limonene from orange extract was shown to significantly increase mouse motor activity, suggesting a potential impact of limonene on locomotion^21^. Limonene may also be an effective therapy for individuals with depression. Komori et al. (1995) treated 12 depressive patients with citrus fragrance exposure and observed a significant improvement in Hamilton Rating Scale for Depression (HRSD) scores, in addition to the normalization of neuroendocrine and immune system markers^29^. Taken together, β-myrcene, linalool and limonene demonstrate significant behavioural changes in animal studies and therefore warrant further investigation.

Zebrafish have become an increasingly prevalent animal model demonstrate clear behavioural and endocrine responses amenable to pharmacological manipulation^30^. As a result, numerous studies have used zebrafish to assess the effects of various drugs on anxiety-like behaviour in zebrafish such as ethanol^31,32^, cocaine^33^, nicotine^34,35^, caffeine^36^, and scopolamine^37^. This study examined the impact of acute exposure to β-myrcene, linalool, and limonene at three different concentrations, on boldness, anxiety-like behaviour, and locomotion in zebrafish. Locomotion and anxiety-like behaviour were assessed by the open field test followed by the novel object approach test which quantified boldness behaviour. The open field and novel object approach test have been used extensively in behavioural neuroscience research and are well-validated measures of anxiety-like behaviour and boldness^30,38,39^. Additionally, in order to examine the differences between short-term and long-term effects of the terpenes investigated, we exposed zebrafish to repeated terpene treatment for seven consecutive days and immediately afterward assessed their behaviour in the open field and novel object approach test.

## Methods

### Animals and housing

Zebrafish (*Danio rerio,* n = 164) were bred in the animal colony at MacEwan University and housed at a maximum density of 15 fish in 10 L tanks within an Aquatic Habitats (AHAB, Aquatic Ecosystems, Inc. Apopka, FL, USA) three-shelf bench top system. MacEwan reared fish were from broodstock obtained from the University of Ottawa (Ottawa, ON, Canada) in March, 2018. Zebrafish were from a wild-type strain. Zebrafish were acclimated in the habitat for a minimum period of one week prior to testing and were adults, mixed gender (~ 50:50, male:female), and experimentally naïve. Daily checks on water pH, dissolved oxygen, and temperature were conducted. The pH of the habitat water was kept between 6.5 and 8.0, dissolved oxygen was 5.0–10.0 ppm, and the temperature was maintained between 26 and 28 °C. The habitat photoperiod was set to an automated 12-h light and dark cycle with the lights turning on at 8AM and off at 8PM. Zebrafish were fed (GEMMA Micro, Westbrook, Maine, United States of America) once per day around noon and after behavioural testing on test days. All experiments were approved by the MacEwan University Animal Research Ethics Board (AREB) under protocol number 05-12-13 in compliance with the Canadian Council for Animal Care (CCAC) guidelines for the care and use of experimental animals. This study was carried out in compliance with the ARRIVE guidelines for animal research.

### Terpene administration

Limonene (97%), β-Myrcene (sum of isomers, ≥ 90%), and Linalool (97%) were purchased from Sigma Aldrich (Ontario, Canada). Terpene solutions were made each testing day by adding the appropriate amount of terpene to 400 ml of water taken directly from the aquatic habitat (i.e. habitat water) in a 600 ml beaker. Solutions were stirred vigorously to mix the terpene with the habitat water. Prior to mixing the habitat water and the respective terpene, habitat water pH was measured. After the addition of the terpene, the pH was measured again. pH of habitat water and terpene solutions were always within 6.8–7.5. Researchers were not blinded to treatment groups but all fish were tested in an identical manner and all data was analyzed with a motion-tracking software system.

### Acute administration

Zebrafish were randomly assigned to either a control group, or to one of the terpene conditions (limonene, linalool, and β-myrcene). Within each terpene condition, fish were further distributed to one of three concentrations. The experimental groups were as follows: limonene 0.25% (n = 15), 0.5% (n = 16), 0.75% (n = 16); linalool 0.0001% (n = 15), 0.001% (n = 15), 0.00125% (n = 15); and β-myrcene 0.001% (n = 15), 0.01% (n = 15), and 0.1% (n = 15). To the best of our knowledge, our study is the first to test terpenes using zebrafish as an animal model, therefore the terpene concentrations were based on previous murine studies^18,22,26^. However, careful pilot testing was performed in order to determine appropriate concentrations of the three terpenes that zebrafish could tolerate. During preliminary testing, we noticed the zebrafish were highly sensitive to linalool and would display signs of extreme sedation (lack of swimming, floating sideways, very immobile). As a result, we used much lower doses of linalool than prior studies^22^.

On testing days, zebrafish were netted from their housing tank and placed in a 600 ml dosing beaker that contained the respective terpene solution. Seedling heat mats (Hydrofarm Horticultural Products, Petaluma CA) were placed underneath the dosing beaker in order to maintain water temperature within 26–28 °C. Zebrafish remained in the dosing beaker for a period of 10 min. In order to prevent external visual stimulation, a piece of white plastic was placed around the beaker^40^. After the 10-min dosing period, fish were immediately netted and placed into the arena for behavioural testing. Identical procedures were performed for control fish excluding the addition of terpene to the dosing beaker.

### Repeated administration

Zebrafish were randomly assigned to one of the following groups, control (n = 20), limonene (0.39%, n = 20), or β-myrcene (0.0083%, n = 20), with each condition containing two replicate groups. The repeated dosing concentrations were based on dose response curve EC_50_ values obtained in the preliminary dosing phase. Linalool was excluded from the repeated dosing experiment due to its limited effect in acute administration studies. Fish were transferred into spawning inserts that were added to the habitat tanks, in order to more efficiently transfer the zebrafish from the habitat tanks to the dosing tanks^32,41^. Dosing tanks contained 1500 ml of habitat water mixed with either 5.775 ml of limonene or 0.125 ml of β-myrcene. Control dosing tanks contained only 1500 ml of habitat water. Habitat tanks were placed adjacent to the dosing tanks for each condition and were separated by white plastic cut-outs in order to prevent any external visual stimulation. The spawning inserts were then removed from the habitat tanks and placed in the dosing tank for 10 min^32,41^. New spawning inserts were then added to the habitat tanks in order to prevent terpene contamination of the habitat tanks. Immediately after the 10-min dosing period, fish were transferred from the old spawning inserts into the habitat tank containing the new spawning insert and placed back into the aquatic habitat three-shelf benchtop system. This process was then repeated for seven days. After dosing on the seventh day, the fish were then netted and placed individually into the arena for behavioural testing. The behavioural testing procedures for the repeated dosing experiment were identical to that of the acute dosing experiment.

### Open field test

The open field test is a commonly used paradigm in behavioural research to assess zebrafish anxiety-like behaviour and locomotion^30,39,42^. In this test, anxiety-like behaviour is quantified as time spent in the various ‘virtual’ zones of the arena (thigmotaxis, transition, and center zone), with more time being spent near the walls of the arena in the thigmotaxis zone being indicative of anxiety-like behaviour. Moreover, a greater amount of time spent in the center zone indicates decreased anxiety-like behaviour. The zones were created within EthoVision XT (v. 11, Noldus, VT, USA) motion tracking software and include a ‘center’ zone that has a diameter of 8.5 cm, a thigmotaxis zone which spanned 8.5 cm from the walls of the arena, and a ‘transition’ zone in between the two (Supp. Fig. 1). Locomotion in this test was quantified by measuring various aspects of movement including distance moved, immobility, high mobility, and meandering. Meandering is a proxy for erratic movement^43^ and was quantified as the change in directional degree per centimeter travelled. Immobility was set to a 5% threshold in EthoVision, meaning that less than a 5% change in the pixels of the detected fish would deem the fish as immobile. Furthermore, fish that demonstrated a minimum 60% change in pixels were deemed highly mobile. Testing took place in a white circular plastic arena with a diameter of 34 cm and a height of 16 cm. For each trial, the arena was filled to a height of 6 cm with fresh habitat water that was changed after every third trial. Trials began after the 10-min dosing period, or last day of repeated administration, and fish were netted and individually placed in the testing arena halfway between the center and thigmotaxis zone. Motion-tracking recording of the fish then began, and trials lasted a duration of 10 min. Additionally, ‘heatmaps’ were generated in EthoVision and display a coloured representation of combined fish localization across all trials within an experimental condition using the ‘per heatmap’ option which generates the colour range based on the data within each group. Due to the sensitivity of the heatmaps to outliers, fish that demonstrated immobility of greater than 100 s in the open field or novel object approach test were removed solely from the group heatmaps. Based on this criteria, n = 1 (myrcene 0.001%), n = 4 (limonene 0.5%), n = 5 (limonene 0.75%), and n = 2 (control) fish were removed from the open field test group heatmaps.

### Novel object approach test

The novel object approach test is another well-validated behavioural assay used to assess boldness and anxiety-like behaviour^30,35,37,39^. Immediately after an open field test trial, a novel object was placed directly in the center of the arena and motion-tracking recording was initiated via EthoVision. The novel object was a LEGO ® figurine (2 cm × 4.25 cm) that was multicoloured in order to prevent any colour bias^35,44^. Trials ran for a duration of 10 min and afterward fish were netted and placed back into their habitat tanks. In each trial, distance moved, time spent in the thigmotaxis, transition, and center zone was measured. The virtual zones in this test were identical to those used in the open field test and group heatmaps were also generated for novel object approach test data. Identical to the open field test, fish who were immobile for 100 s or greater during a novel object approach trial were removed solely from group heatmaps. This includes n = 2 (myrcene 0.001%), n = 2 (limonene 0.25%), n = 4 (limonene 0.5%), n = 2 (linalool 0.00125%), and n = 3 (control).

### Statistical analysis

Data were assessed for normality using the D’Agostino-Pearson omnibus normality test. For the acute dosing experiment, parametric data was analyzed using a one-way ANOVA followed by post-hoc Dunnett’s multiple comparison test. Non-parametric data in the acute dosing experiment was analyzed using a Kruskal–Wallis with post-hoc Dunn’s multiple comparison test. For the repeated dosing experiment parametric data was analyzed using a nested one-way ANOVA with Dunnett’s multiple comparison test. Non-parametric data was first normalized by setting the smallest value in the data set as 0%, and the largest value as 100%, then a nested one-way ANOVA with post-hoc Dunnett’s multiple comparison test was performed. Additionally, multiple comparison tests in both experiments had the significance level adjusted using multiplicity adjusted P values. All data were analyzed using GraphPad Prism (Version 9.1.0; GraphPad, San Diego, CA, USA). EC_50_ values were calculated in Prism using the time in the thigmotaxis zone in the novel object approach test as the dependent variable. The percent concentrations for each terpene used in the preliminary testing phase were first converted to their molar concentrations. Subsequently, the molar concentrations were transformed into their corresponding log values by the log(x) transform function in Prism. Lastly, the y-value data, or the time in the thigmotaxis zone data, was normalized by setting the highest value in each data set as 100% and the lowest as 0%, and a non-linear regression analysis was then performed in order to obtain the EC_50_ value. An alpha level of *P* < 0.05 and a 95% confidence interval was used to indicate statistical significance. All values are presented as mean ± S.E.M.

## Results

### Acute dosing experiment

#### Limonene—open field test

**Figure 1:**
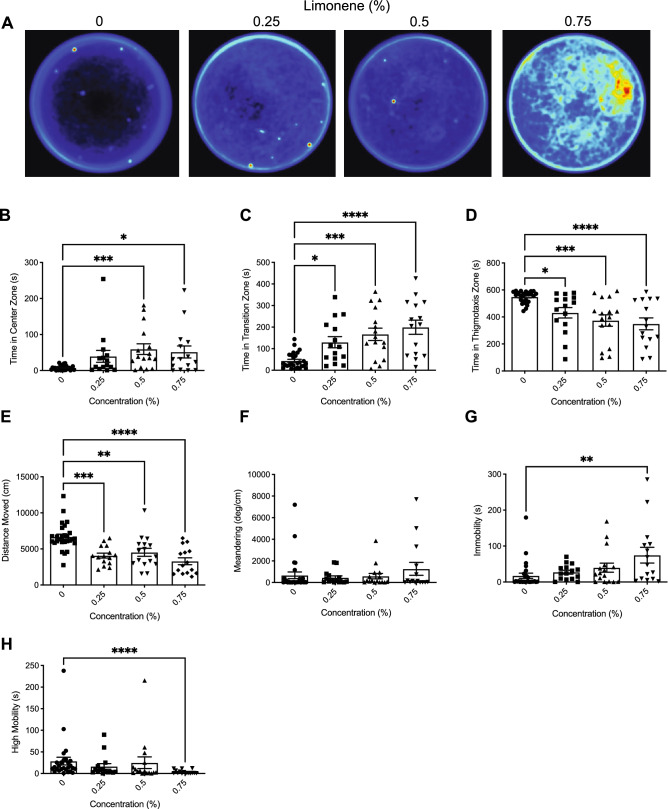
The effects of acute limonene administration on zebrafish behaviour assessed by the open field test. (**A**) Group heatmaps showing a coloured representation of the combined mean location of each group of fish across all trials. Average time spent in the center (**B**), transition (**C**), and thigmotaxis (**D**) zone during the open field test. Fish locomotion was quantified in the open field test by measuring distance moved (**E**), meandering (**F**), time spent immobile (**G**), and time spent highly mobile (**H**). All data are presented as mean ± S.E.M. Significant differences between controls and limonene treated groups are indicated by *(*P* < 0.05), **(*P* < 0.01), ***(*P* < 0.001), and ****(*P* < 0.0001).

Heatmaps created from video recordings show the combined group behaviour for the control and limonene groups for the open field trials (Fig. 1A).

##### Time in zones

We observed a significant difference between groups for time spent in the center zone (Fig. 1B; *H*(3) = 17.02, *P* = 0.0007). Dunn’s multiple comparison test indicated significant differences compared to controls for treatments 0.5% (*P* = 0.0004) and 0.75% (*P* = 0.0228). Significant differences between groups were also observed for time spent in the transition zone (Fig. 1C; *H*(3) = 23.77, *P* =  < 0.0001). Dunn’s multiple comparison test showed that the 0.25% (*P* = 0.0149), 0.5% (*P* = 0.0008), and 0.75% (*P* =  < 0.0001) treatments were significantly different from controls. The time spent in the thigmotaxis zone was also significantly different between groups (Fig. 1D; *F*(3, 69) = 8.248, *P* =  < 0.0001). Dunn’s multiple comparison test showed that the 0.25% (*P* = 0.0190), 0.5% (*P* = 0.0002), and 0.75% (*P* =  < 0.0001) treatments groups were significantly different from controls.

##### Locomotion

We observed a significant difference in distanced moved between treatments (Fig. 1E; *H*(3) = 29.82, *P* =  < 0.0001). Post-hoc Dunn’s multiple comparison test indicated that there were significant differences compared to controls in distance moved for 0.25% (P = 0.0004), 0.5% (*P* = 0.0026), and 0.75% (*P* =  < 0.0001). No significant differences were observed between groups in meandering (Fig. 1F; *H*(3) = 3.099, *P* = 0.3766). Immobility was also observed to be significantly different between groups (Fig. 1G; *H*(3) = 13.85, *P* = 0.0031). Dunn’s multiple comparison test showed that the 0.75% (*P* = 0.0012) group spent significantly more time immobile than controls. We observed a significant difference between groups in high mobility (Fig. 1H; *H*(3) = 17.91, *P* = 0.0005). Dunn’s multiple comparison test showed that the 0.75% group had decreased high mobility compared to controls (*P* =  < 0.0001).

#### Linalool—open field test

**Figure 2:**
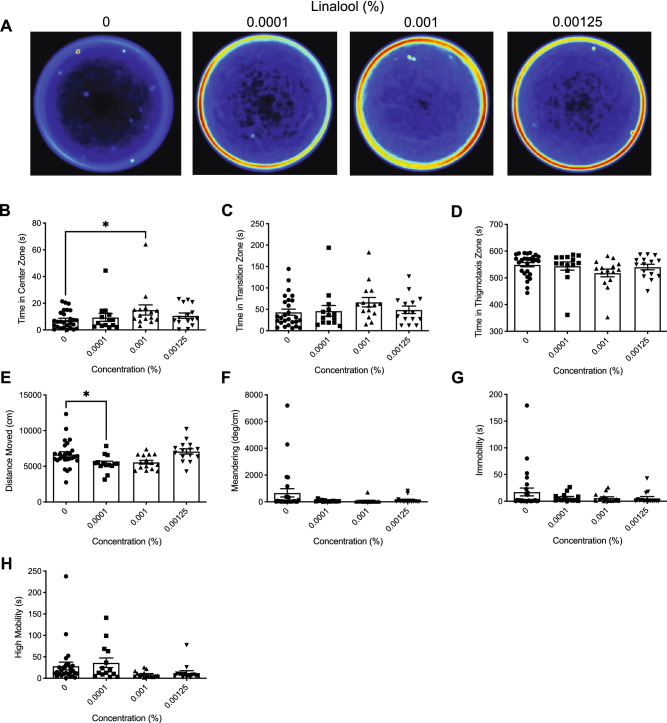
The effects of acute linalool administration on zebrafish behaviour assessed by the open field test. (**A**) Group heatmaps showing a coloured representation of the combined mean location of each group of fish across all trials. Average time spent in the center (**B**), transition (**C**), and thigmotaxis (**D**) zone during the open field test. Fish locomotion was quantified in the open field test by measuring distance moved (**E**), meandering (**F**), time spent immobile (**G**), and time spent highly mobile (**H**). All data are presented as mean ± S.E.M. Significant differences between controls and linalool treated groups are indicated by *(*P* < 0.05).

Heatmaps created from video recordings show the combined group behaviour for the control and linalool groups for the open field trials (Fig. 2A).

##### Time in zones

We observed a significant difference between groups for time spent in the center zone (Fig. 2B; *H*(3) = 7.642, *P* = 0.0540). Dunn’s multiple comparison test indicated that the 0.001% group spent significantly more time in the center zone than controls (*P* = 0.0286). There were no significant differences between groups for time spent in the transition (Fig. 2C; *H*(3) = 4.963, *P* = 0.1745). Moreover, no significant differences were observed for time spent in the thigmotaxis zone (Fig. 2D; *H*(3) = 5.624, *P* = 0.1314).

##### Locomotion

Here, we observed a significant difference in distance moved between groups (Fig. 2E; *H*(3) = 15.54, *P* = 0.0014). Dunn’s multiple comparison test indicated that the 0.0001% (*P* = 0.0311) linalool treatment group had travelled a significantly less than controls. We observed no significant differences between groups in meandering (Fig. 2F; *H*(3) = 4.810, *P* = 0.1862), and immobility (Fig. 2G; *H*(3) = 5.165, *P* = 0.1601). We did observe a significant difference between groups in high mobility (Fig. 2H; *H*(3) = 10.32, *P* = 0.0160). However, Dunn’s multiple comparison test did not indicate any significant differences for treatment groups compared to controls.

#### Myrcene—open field test

**Figure 3:**
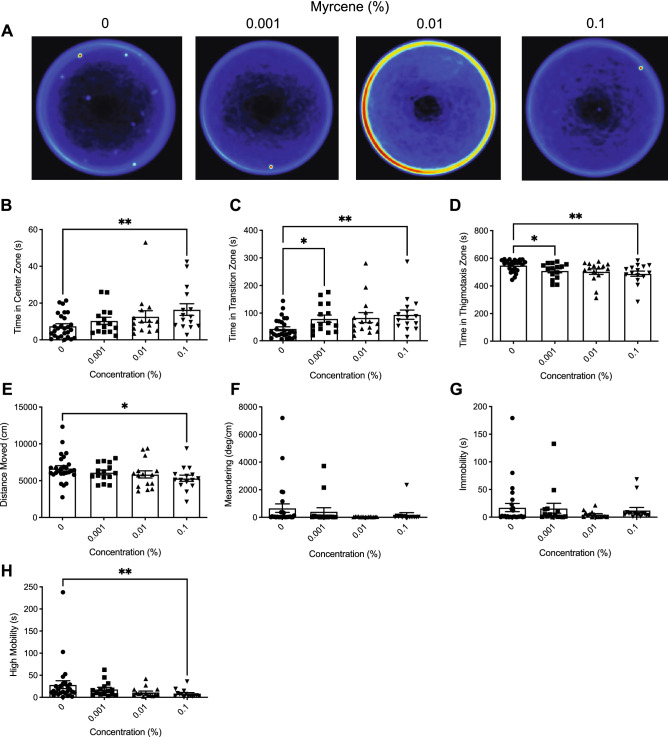
The effects of acute β-myrcene administration on zebrafish behaviour assessed by the open field test. (**A**) Group heatmaps showing a coloured representation of the combined mean location of each group of fish across all trials. Average time spent in the center (**B**), transition (**C**), and thigmotaxis (**D**) zone during the open field test. Fish locomotion was quantified in the open field test by measuring distance moved (**E**), meandering (**F**), time spent immobile (**G**), and time spent highly mobile (**H**). All data are presented as mean ± S.E.M. Significant differences between controls and β-myrcene treated groups are indicated by *(*P* < 0.05) and **(*P* < 0.01).

Heatmaps created from video recordings show the combined group behaviour for the control and myrcene groups for the open field trials (Fig. 3A).

##### Time in zones

We observed significant difference between groups in time spent in the center zone (Fig. 3B; *H*(3) = 9.587, *P* = 0.0225). Dunn’s multiple comparison test indicated that the 0.1% treatment group spent significantly more time in the center zone then controls (*P* = 0.0083). Additionally, we observed significant differences for time spent in the transition zone (Fig. 3C; *H*(3) = 13.78, *P* = 0.0032). Dunn’s multiple comparison test indicated that the 0.001% and 0.1% treatment groups spent significantly more time in the transition zone (*P* = 0.0365 and *P* = 0.0028 respectively) than controls. Significant differences between groups were also seen for time spent in the thigmotaxis zone (Fig. 3D; *H*(3) = 13.87, *P* = 0.0031). Dunn’s multiple comparison test indicated that the 0.001% and 0.1% treatments groups spent significantly less time in the thigmotaxis zone than controls (*P* = 0.0426 and *P* = 0.0023 respectively).

##### Locomotion

We observed a significant difference between groups in distance moved (Fig. 3E; *H*(3) = 8.307, *P =* 0.0401). Dunn’s multiple comparison test indicated that the 0.1% treatment travelled significantly less than controls (*P* = 0.0190). No significant differences were observed between groups in meandering (Fig. 3F; *H*(3) = 1.484, *P* = 0.6860) or immobility (Fig. 3G; *H*(3) = 3.824, *P* = 0.2811). However, we did observe a significant difference between groups in high mobility (Fig. 3H; *H*(3) = 10.47, *P* = 0.0150). Dunn’s multiple comparison test indicated that the 0.1% treatment group had significantly shorter duration of time spent high mobile than controls (*P* = 0.0094).

#### Limonene—novel object approach test

**Figure 4:**
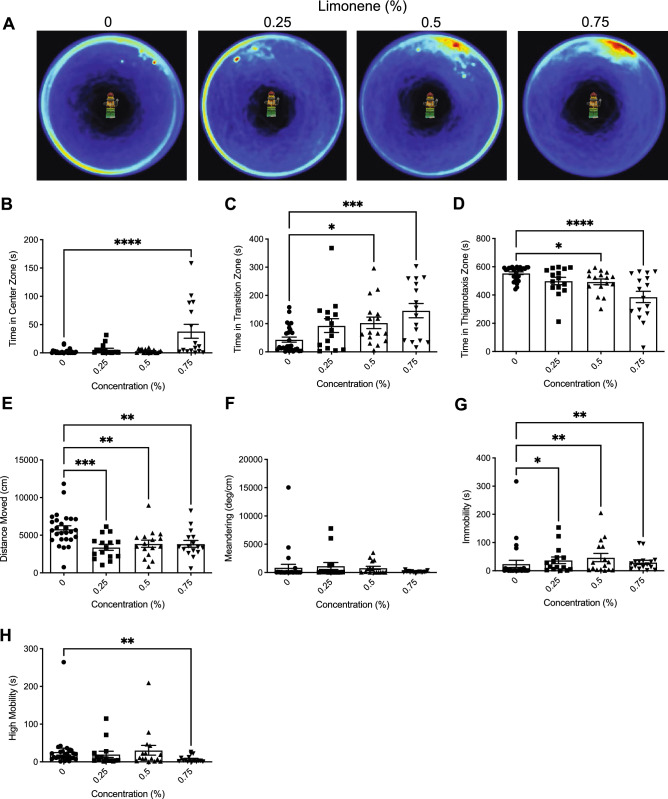
The effects of acute limonene administration on zebrafish behaviour assessed by the novel object approach test. (**A**) Group heatmaps showing a coloured representation of the combined mean location of each group of fish across all trials. Average time spent in the center (**B**), transition (**C**), and thigmotaxis (**D**) zone during the novel object approach test. Fish locomotion was quantified in the open field test by measuring distance moved (**E**), meandering (**F**), time spent immobile (**G**), and time spent highly mobile (**H**). All data are presented as mean ± S.E.M. Significant differences between controls and limonene treated groups are indicated by *(P < 0.05), **(*P* < 0.01), *** (*P* < 0.001), and ****(*P* < 0.0001).

Heatmaps created from video recordings show the combined group behaviour for the control and limonene groups for the novel object approach test trials (Fig. 4A).

##### Time in zones

We observed a significant difference between groups in time spent in the center zone (Fig. 4B; *H*(3) = 18.03, *P* = 0.0004). Dunn’s multiple comparison test showed that the 0.75% treatment spent significantly more time in the center than controls (*P* = 0.0001). Significant differences were also observed in time spent in the transition zone (Fig. 4C; *H*(3) = 15.58, *P* = 0.0014). Dunn’s multiple comparison test showed that the 0.5% (*P* = 0.0318) and 0.75% (*P* = 0.0006) treatments spent significantly more time in the transition zone than controls. Furthermore, we observed a significant difference in time spent in the thigmotaxis zone between groups (Fig. 4D; *H*(3) = 19.37, *P* = 0.0002). Dunn’s multiple comparison test showed that the 0.5% (*P* = 0.0369) and 0.75% (*P* =  < 0.0001) treatments spent significantly less time in the thigmotaxis zone than controls.

##### Locomotion

We observed significant differences in distance moved between treatments (Fig. 4E; *H*(3) = 19.14, *P* = 0.0003). Dunn’s multiple comparison test indicated that the 0.25% (*P* = 0.0007), 0.5% (*P* = 0.0064), and 0.75% (*P* = 0.0048) treatments travelled significantly less than controls. No significant differences between groups were observed in meandering (Fig. 4F; *H*(3) = 4.722, *P* = 0.1933). However, we did observe significant differences between groups in immobility (Fig. 4G; *H*(3) = 16.48, *P* = 0.0009). Dunn’s multiple comparison test showed that the 0.25% (*P* = 0.0144), 0.5% (*P* = 0.0062), and 0.75% (*P* = 0.0028) treatments spent significantly more time immobile than controls. Additionally, we observed a significant difference between treatments in high mobility (Fig. 4H; *H*(3) = 10.61, *P* = 0.0141). Dunn’s multiple comparison test indicated that the 0.75% treatment spent significantly less time highly mobile than controls (*P* = 0.0040).

#### Linalool—novel object approach test

**Figure 5:**
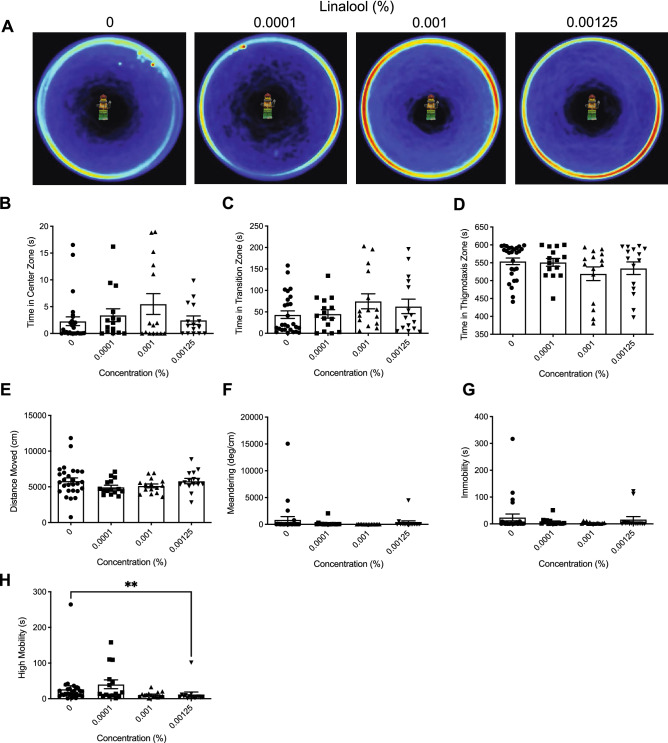
The effects of acute linalool administration on zebrafish behaviour assessed by the novel object approach test. (**A**) Group heatmaps showing a coloured representation of the combined mean location of each group of fish across all trials. Average time spent in the center (**B**), transition (**C**), and thigmotaxis (**D**) zone during the novel object approach test. Fish locomotion was quantified in the open field test by measuring distance moved (**E**), meandering (**F**), time spent immobile (**G**), and time spent highly mobile (**H**). All data are presented as mean ± S.E.M. Significant differences between controls and linalool treated groups are indicated by **(*P* < 0.01).

Heatmaps created from video recordings show the combined group behaviour for the control and linalool groups for the novel object approach test trials (Fig. 5A).

##### Time in zones

Here we observed no significant differences between groups in time spent in center zone (Fig. 5B; *H*(3) = 1.803, *P* = 0.6144), transition zone (Fig. 5C; F(3,68) = 1.324, *P* = 0.2736), or thigmotaxis zone (Fig. 5D; F(3,68) = 1.375, *P* = 0.2578.

##### Locomotion

Here, we observed no significant differences between groups in distance moved (Fig. 5E; *H*(3) = 5.052, *P* = 0.1680), meandering (Fig. 5F; *H*(3) = 5.010, *P* = 0.1711), or immobility (Fig. 5G; *H*(3) = 2.977, *P* = 0.3952). However, we did observe a significant difference between groups in high mobility (Fig. 5H; *H*(3) = 15.08, *P* = 0.0017). Dunn’s multiple comparison test showed that the 0.00125% treatment spent significantly less time highly mobile than controls (*P* = 0.0033).

#### Myrcene—novel object approach test

**Figure 6:**
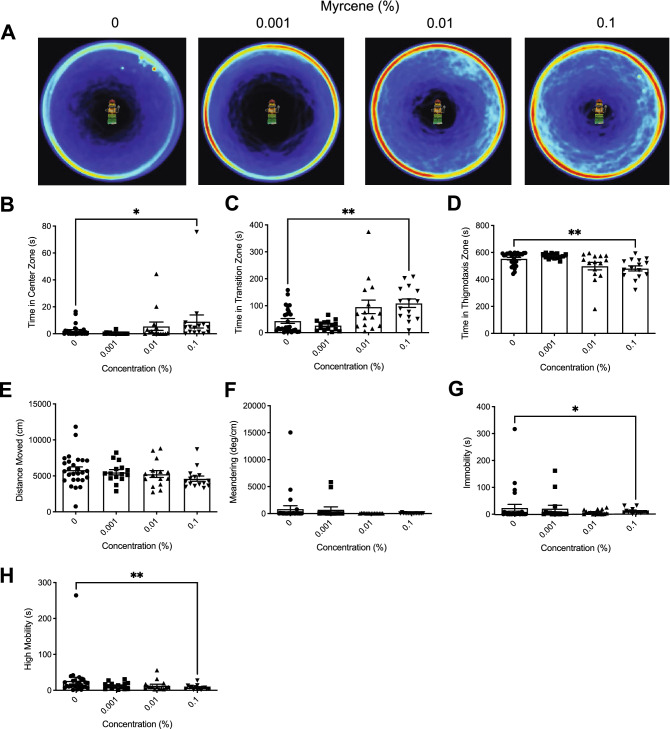
The effects of acute β-myrcene administration on zebrafish behaviour assessed by the novel object approach test. (**A**) Group heatmaps showing a coloured representation of the combined mean location of each group of fish across all trials. Average time spent in the center (**B**), transition (**C**), and thigmotaxis (**D**) zone during the novel object approach test. Fish locomotion was quantified in the novel object approach test by measuring distance moved (**E**), meandering (**F**), time spent immobile (**G**), and time spent highly mobile (**H**). All data are presented as mean ± S.E.M. Significant differences between controls and β-myrcene treated groups are indicated by *(*P* < 0.05) and **(*P* < 0.01).

Heatmaps created from video recordings show the combined group behaviour for the control and myrcene groups for the novel object approach test trials (Fig. 6A).

##### Time in zones

Significant differences were observed between groups for time spent in the center (Fig. 6B; *H*(3) = 17.04, *P* = 0.0007), transition (Fig. 6C; *H*(3) = 19.33, *P* = 0.0002) and thigmotaxis zone (Fig. 6D; *H*(3) = 19.45, *P* = 0.0002). Dunn’s multiple comparison test indicated that compared to controls, the 0.1% treatment spent significantly more time in the center (*P* = 0.0460) and transition zone (*P* = 0.0014) and significantly less time in the thigmotaxis zone (*P* = 0.0016).

##### Locomotion

No significant differences were observed between groups in distance moved (Fig. 6E; *H*(3) = 6.675, *P* = 0.0830). No significant differences between treatments were observed in meandering (Fig. 6F; *H*(3) = 1.462, *P* = 0.6910). However, significant differences between treatments were observed in immobility (Fig. 6G; *H*(3) = 8.327, *P* = 0.0397) and high mobility (Fig. 6H; *H*(3) = 11.15, *P* = 0.0109). Dunn’s multiple comparison test indicated that compared to controls, the 0.1% treatment group spent significantly less time immobile (*P* = 0.0134) as well as significantly less time highly mobile (*P* = 0.0033).

### Repeated dosing experiment

Because of the robust anxiolytic effects of β-myrcene and limonene in our acute administration study, we tested the effect or repeated administration of these compounds. First, we calculated EC_50_ values of β-myrcene (0.0083%) and limonene (0.39%) and used these concentrations for the repeated dosing experiment.

#### Open field test

##### Time in zones

**Figure 7:**
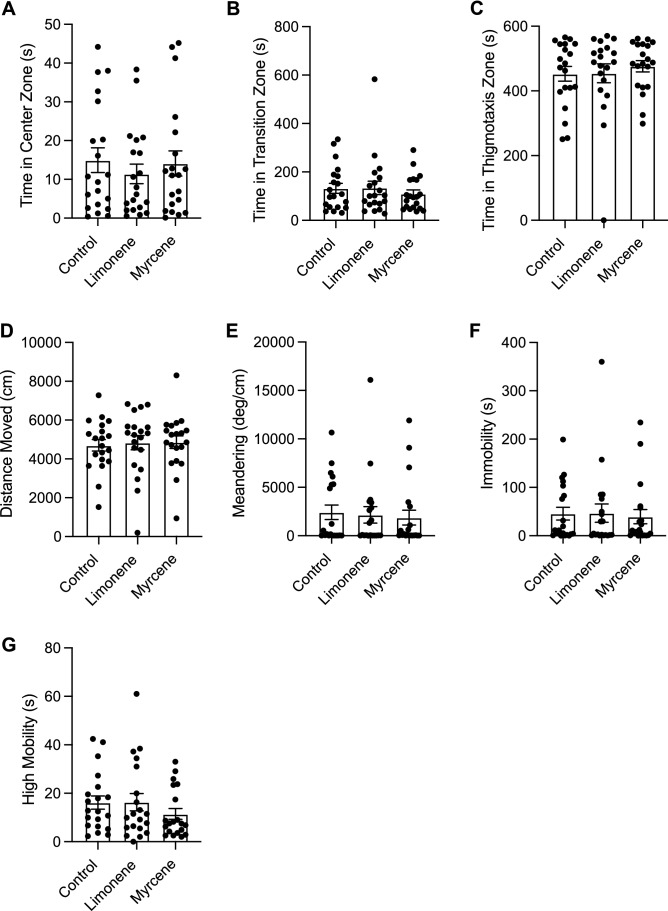
The effects of repeated terpene administration (limonene or β-myrcene) on zebrafish behaviour assessed by the open field test. Average time spent in the center (**A**), transition (**B**), and thigmotaxis (**C**) zone during the open field test. Fish locomotion was quantified in the open field test by measuring distance moved (**D**), meandering (**E**), time spent immobile (**F**), and time spent highly mobile (**G**). All data are presented as mean ± S.E.M. No significant differences were observed between control and terpene treated groups.

Here, we observed no significant differences between any group in time spent in the center zone (Fig. 7A; F(2,3) = 0.1441, *P* = 0.8714). Moreover, no significant differences were seen in time spent in the transition zone (Fig. 7B; F(2, 57) = 0.3813, *P* = 0.6847) or time spent in the thigmotaxis zone (Fig. 7C; F(2, 57), *P* = 0.3043).

##### Locomotion

We observed no significant differences between groups in distance moved (Fig. 7D; F(2, 57) = 0.07074, *P* = 0.9318). Additionally, there were no significant differences between groups in meandering (Fig. 7E; F(2, 57) = 0.1237, *P* = 0.8839), immobility (Fig. 7F; F(2, 57) = 0.06465, *P* = 0.9375), or high mobility (Fig. 7G; F(2,57) = 0.9144, *P* = 0.4065).

#### Novel object approach test

##### Time in zones

**Figure 8:**
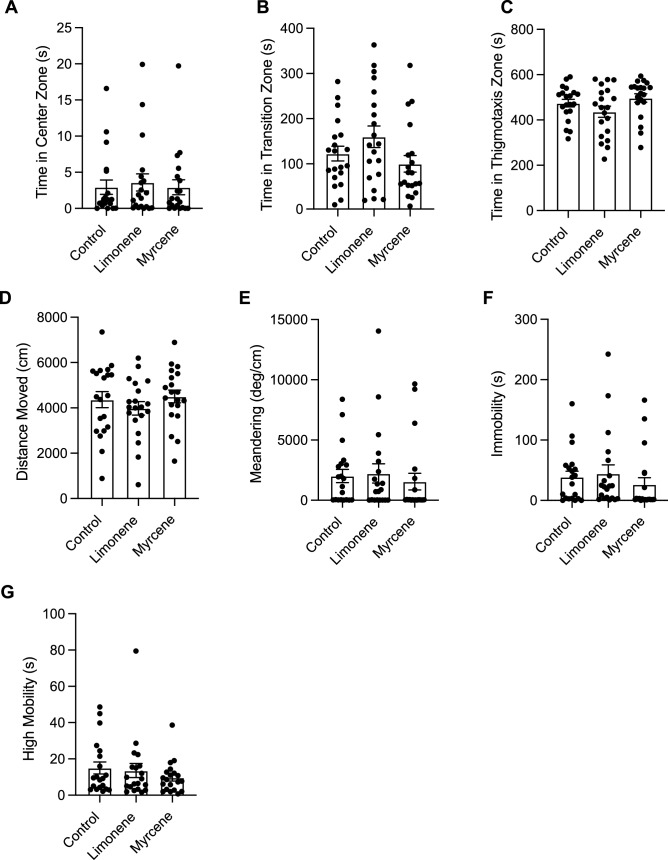
The effects of repeated terpene administration (limonene or β-myrcene) on zebrafish behaviour assessed by the novel object approach test. Average time spent in the center (**A**), transition (**B**), and thigmotaxis (**C**) zone during the novel object approach test. Fish locomotion was quantified in the novel object approach test by measuring distance moved (**D**), meandering (**E**), time spent immobile (**F**), and time spent highly mobile (**G**). All data are presented as mean ± S.E.M. No significant differences were observed between control and terpene treated groups.

There were no significant differences between groups in time spent in the center zone (Fig. 8A; F(2, 3) = 0.07236, *P* = 0.9318). No significant differences were seen in time spent in the transition zone (Fig. 8B; F(2, 57) = 2.326, *P* = 0.1069). Furthermore, we observed no significant differences in time spent in the thigmotaxis zone (Fig. 8C; F(2, 57) = 2.326, *P* = 0.1069).

##### Locomotion

Here, we observed no significant differences between groups in distance moved (Fig. 8D; F(2, 57) = 0.7433, *P* = 0.4801). Moreover, there were no significant differences between treatments in meandering (Fig. 8E; F(2, 3) = 0.2369, *P* = 0.8025), immobility (Fig. 8F; F(2, 57) = 0.5978, *P* = 0.5534), or high mobility (Fig. 8G; F(2, 3) = 0.4706, *P* = 0.6641).

## Discussion

This study investigated the effect of terpenes on zebrafish locomotion, anxiety-like behaviour, and boldness when dosed acutely, and repeatedly over a 7-day period. This yielded three major findings. First, in the acute dosing experiment limonene and β-myrcene had significant effects on a myriad of variables that confer decreased anxiety-like behaviour, increased boldness, and altered locomotion. Second, zebrafish exposed to 0.0001% linalool demonstrated a significant reduction in locomotion in the open field test, suggesting a sedative effect at this concentration. Lastly, locomotion, anxiety-like behaviour, boldness, and locomotion were not impacted by repeated exposure to limonene or β-myrcene for 7 consecutive days, suggesting that repeated exposure causes neuroadaptations or metabolic tolerance resulting in a negligible effect on behaviour.

### Acute dosing experiment

In the open field test, we observed reductions in locomotion in all three terpenes tested (see Figs. 1, 2, and 3). This is consistent with previous murine studies which noted a similar decrease in locomotion as a result of limonene, β-myrcene, and linalool administration. Do Vale et al. (2002) observed that a 200 mg/kg dose of limonene significantly decreased the number of crossings in the arena by 71% and rearing behaviour at doses of 100 and 200 mg/kg by 55–88% in an open field test^18^. β-Myrcene showed similar effects with a 100 and 200 mg/kg dose decreasing the number of crossings by 49 and 36% and rearing behaviour by 70 and 79% respectively^18^. Interestingly, β-myrcene and limonene were shown to have similar effects to diazepam (a benzodiazepine with sedative properties) on locomotion in an open field test^18^. Similarly, de Almeida et al. (2012) also found significant decreases in murine locomotion that was comparable to diazepam in an open field test from 25, 50, and 75 mg/kg intraperitoneal injections of limonene^27^. In our study, in contrast to limonene and β-myrcene, linalool caused less of a decrease on locomotion. Distance moved was decreased compared to controls only at the lowest dose (Fig. 2e, f; 0.0001%) while meandering, immobility, and high mobility, were not impacted (Fig. 2g, h, and i respectively). The decrease in distance moved observed at 0.0001% is consistent with Buchbauer et al. (1993), who found that mice exposed to a linalool fragrance had a 73% reduction in motility^21^. However, it is unknown why higher concentrations did not have the same effect. Overall, limonene and β-myrcene produced sedative effects on zebrafish, consistent with previous literature.

Limonene, myrcene, and linalool also demonstrated significant reductions in anxiety-like behaviour in the open field test (Fig. 1b–d, Fig. 2b, and Fig. 3b–d). Limonene (0.5% and 0.75%), linalool (0.001%), and β-myrcene (0.1%) groups all spent significantly more time in the center zone, while limonene (0.25%, 0.5%, and 0.75%) and β-myrcene (0.001% and 0.1%) spent significantly less time in the thigmotaxis zone compared to controls. This finding is consistent with murine studies that observed anxiolytic effects from linalool inhalation assessed by the light/dark test^23^ and from limonene inhalation assessed by the elevated plus maze test^26^. In contrast to the anxiolytic effect of β-myrcene found in our study, do Vale et al. (2002) observed that mice injected with 25 mg/kg of β-myrcene had a significant reduction of number of entries to the open arms of the elevated plus maze test, indicating an anxiogenic effect^18^. This discrepancy could be due to a multitude of factors including species differences, route of administration, and chosen terpene concentrations. For instance, do Vale et al. (2002) also found no significant differences in mice behaviour for intraperitoneal injection of limonene at concentrations of 10, 25, and 50 mg/kg compared to controls in the elevated plus test^18^. However, Lima et al. (2013) found that mice exposed to 0.5% and 1% inhaled limonene spent a significantly greater amount of time in the open arms of an elevated plus maze test as well as increased number of entries in the open arms compared to controls, indicating an anxiolytic effect^26^. Thus, the impact of terpenes on behaviour may be sensitive to the concentrations utilized and the route of administration.

The novel object approach test is a reliable behavioural paradigm that measures exploratory behaviour in the presence of a novel object. Boldness in animals may lead to benefits such as foraging opportunities, increasing mate attraction, and gaining information about a predator threat^45^. Thus, there exists a delicate balance of risk versus reward, where ‘bold’ fish are more likely to engage in novel object inspection. In our study, we observed a significant increase in boldness in the novel object approach test for limonene and β-myrcene. At certain concentrations (See Fig. 4b–d and Fig. 6b–d), zebrafish exposed to limonene (0.5 and 0.75%) or β-myrcene (0.1%) demonstrated an increase in time spent in the center zone and transition zone, and a decrease in time spent in the thigmotaxis zone, which is consistent with increased boldness. Interestingly, linalool did not have a significant impact on zebrafish boldness in the novel object approach test at any concentration. Linck et al. (2010) found that mice exposed to a 1% linalool treatment demonstrated increased social interaction and decreased aggression when presented when paired with an unfamiliar mouse^23^, which could be interpreted as an increase in boldness facilitating socialization. Possibly our behavioural test is not as sensitive as a social interaction measure? However, it has been shown that when zebrafish are kept in pairs, bold zebrafish are more likely to act aggressively in order to establish dominant-subordinate relationships^46^. Laboratory mice have also demonstrated the tendency to develop a social hierarchy that is maintained through aggression^47^. Thus, if linalool exposure has a similar effect on mice and zebrafish, decreased aggression would suggest less bold behaviour rather than increased boldness, and therefore future studies should examine exploratory behaviour with other paradigms.

### Repeated dosing experiment

In the acute dosing experiment, we observed numerous significant behavioural alterations with β-myrcene and limonene, however, in the repeated dosing experiment we observed no significant effects limonene or β-myrcene on anxiety-like behaviour, boldness, and locomotion. This lack of behavioural response may be due to a variety of reasons. Zebrafish may become quickly tolerant to terpenes resulting in no behavioural effects when tested at the end of the repeated dosing schedule. Prior zebrafish studies on repeated drug exposure and withdrawal have shown that the timing of testing after a repeated dosing schedule can have a significant impact on the results. Dean et al. (2020) repeatedly dosed zebrafish with nicotine for a period of 21 days and assessed behaviour using the novel object approach test immediately after the 21-day period and again on day 23^35^. Dean et al. (2020) observed no significant changes to locomotion when tested on day 21, however locomotion was significantly decreased when tested again on day 23, suggesting symptoms of nicotine withdrawal took time to manifest^35^. Moreover, Dean et al. (2020) observed opposite effects in boldness behaviour between zebrafish acutely exposed to nicotine and those exposed repeatedly over a 21-day period^35^. Zebrafish acutely exposed to nicotine demonstrated an increase in boldness behaviour, whereas zebrafish chronically exposed to nicotine for a 21 day period and tested one hour afterward demonstrated a decrease in boldness^35^. This discrepancy between acute and repeated exposure results is congruent with our study which found significant effects in the acute dosing experiment but no significant effects in the repeated dosing experiment. This same pattern is present in the results from Holcombe et al. (2013), who observed significant increase in anxiety-like behaviour in zebrafish exposed to a daily 0.2% dose of ethanol when tested on day 2 of withdrawal, but not day 9^48^. In our study, results suggest that there were no effects of repeated limonene or β-myrcene exposure in zebrafish anxiety-like behaviour, boldness or locomotion. This could be due to pharmacodynamic factors (i.e. neuroadaptation), pharmacokinetic factors (i.e. increased metabolism), or a combination of the two. Because we only tested at one time point which was immediately after the 7-day dosing period, it is possible that we failed to capture the true effects of repeated limonene or β-myrcene exposure and testing two days after the dosing period could yield a withdrawal effect. Future behavioural studies would benefit from withdrawal testing to better understand the development of tolerance to these compounds.

In conclusion, our findings indicate that zebrafish acutely dosed with limonene and β-myrcene exhibit decreased anxiety-like behaviour and locomotion and increased boldness. Linalool had little effect on anxiety-like and boldness behaviour and mainly impacted locomotion. Additionally, repeated dosing with limonene and β-myrcene produced no effects compared to controls, suggesting that zebrafish quickly build tolerance to these compounds. Our study combined with previous research provides strong support for a sedative and anxiolytic effect of limonene and β-myrcene, and warrants further investigation into the therapeutic value of terpenes in animal models and in humans. Terpenes have the potential to become valuable therapeutic compounds for the treatment of many mental health conditions.

## Supplementary Information

Supplementary Information 1.

Supplementary Information 2.

Supplementary Information 3.

Supplementary Information 4.
