# Supplementary material for: Robust behavioural effects in response to acute, but not repeated, terpene administration in Zebrafish (Danio rerio)
Source: Sci Rep. 2021 Sep 28;11:19214. doi: 10.1038/s41598-021-98768-1 (PMC8478887; doi:10.1038/s41598-021-98768-1)
Supplement: Supplementary file 4 — Supplementary Information 4. [file 41598_2021_98768_MOESM4_ESM.docx]

**Title: Robust behavioural effects in response to acute, but not repeated, terpene administration in Zebrafish (*Danio rerio*)**

Joshua Szaszkiewicz^1^, Shannon Leigh^1^, Trevor J. Hamilton^1,2^*

^1^Department of Psychology, MacEwan University, Edmonton, AB, Canada, T5J 4S2.

^2^Neuroscience and Mental Health Institute, University of Alberta, Edmonton, AB, Canada, T6G 2H7.

**Supplementary Material**


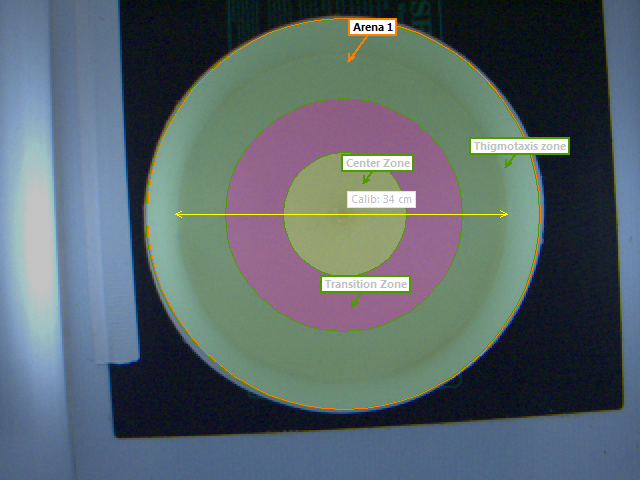


Supplementary Figure 1: Experimental zones used in the open field and novel object approach test. Shown is the image from EthoVision with each zone overlayed on the open field test. The center zone (yellow), transition zone (pink) and thigmotaxis zone (light green) were used to quantify time fish spent in each area. Note that the water is not present in this image. Zones are identical for the novel object approach test.
